# Roles of Mast Cells in Cutaneous Diseases

**DOI:** 10.3389/fimmu.2022.923495

**Published:** 2022-07-06

**Authors:** Takafumi Numata, Kazutoshi Harada, Susumu Nakae

**Affiliations:** ^1^ Department of Dermatology, Tokyo Medical University, Tokyo, Japan; ^2^ Graduate School of Integrated Sciences for Life, Hiroshima University, Hiroshima, Japan; ^3^ Precursory Research for Embryonic Science and Technology, Japan Science and Technology Agency, Saitama, Japan

**Keywords:** skin disease, allergy, autoimmunity, infection, rejection

## Abstract

Mast cells are present in all vascularized tissues of the body. They are especially abundant in tissues that are in frequent contact with the surrounding environment and act as potential sources of inflammatory and/or regulatory mediators during development of various infections and diseases. Mature mast cells’ cytoplasm contains numerous granules that store a variety of chemical mediators, cytokines, proteoglycans, and proteases. Mast cells are activated *via* various cell surface receptors, including FcϵRI, toll-like receptors (TLR), Mas-related G-protein-coupled receptor X2 (MRGPRX2), and cytokine receptors. IgE-mediated mast cell activation results in release of histamine and other contents of their granules into the extracellular environment, contributing to host defense against pathogens. TLRs, play a crucial role in host defense against various types of pathogens by recognizing pathogen-associated molecular patterns. On the other hand, excessive/inappropriate mast cell activation can cause various disorders. Here, we review the published literature regarding the known and potential inflammatory and regulatory roles of mast cells in cutaneous inflammation, including atopic dermatitis, psoriasis, and contact dermatitis GVHD, as well as in host defense against pathogens.

## Introduction

Mast cells are tissue-resident immune cells that are derived from hematopoietic stem cells (1). First, mast cell progenitors (MCPs) differentiate from hematopoietic stem cells in the bone marrow and/or spleen (shown in mice) and circulate *via* the vascular system (2, 3). The MCPs then infiltrate the local tissues from the blood, where they differentiate into functionally-mature mast cells under the control of the components of the cytokine milieu, such as stem cell factor (SCF), transforming growth factor (TGF)-β, nerve growth factor (NGF), interleukin (IL)-3, IL-4, IL-9, and IL-33 (4–6). Therefore, mast cells are present in all vascularized tissues of the body, and they are especially abundant in tissues that come into frequent contact with the surrounding environment, such as the gastrointestinal tract, skin, and respiratory epithelium (2).

Mature mast cells’ cytoplasm contains numerous granules that store a variety of chemical mediators (e.g., histamine), proteoglycans, and proteases. In both rodents and humans, mast cells have been categorized into two types based on their anatomical distribution and the kinds of proteases stored in their granules. In rodents, one type is the mucosal mast cell (MMC), which are located in the mucosa (mucosal epithelium) and whose granules contain tryptase (2). The second type is the connective tissue-type mast cell (CTMC), which are located in connective tissues such as the skin and submucosa and whose granules contain chymase, carboxypeptidases, and tryptase. In humans, one type is termed the TC mast cell (MC_TC_), whose granules contain tryptase and chymase, while the second type is termed the T mast cell (MC_T_), whose granules contain only tryptase (7).

In response to certain stimuli, mast cells release the contents of their granules into the extracellular environment this process is known as degranulation. In one major pathway, degranulation occurs immediately following crosslinking of antigens by antigen-specific immunoglobulin (Ig) E that is bound to FcϵRI on mast cells (8). Mobilization of Ca^2+^ is a key process that occurs during degranulation of mast cells after antigen/IgE/FcϵRI-crosslinking (9, 10). With or without degranulation, mast cells can also release *de novo*-synthesized inflammatory mediators. In addition, degranulated mast cells are able to replenish their granules, allowing them to undergo repeated degranulation in tissues (11).

Mast cells are able to mount a rapid immunological response by releasing prestored inflammatory mediators, and, owing to their location in the skin and mucosa, they are a part of the front line of defense against pathogens invading the body (12). The released inflammatory mediators bring about increased vascular permeability and fluid accumulation, as well as recruitment and activation of immune cells, including dendritic cells, macrophages, T cells, and B cells (2, 13). However, excessive mast cell activation can rapidly cause death *via* anaphylactic shock. Moreover, inappropriate mast cell activation can cause various diseases such as allergic and autoimmune diseases (14). On the other hand, mast cells play a suppressive role in certain diseases. Thus, they can act not only as pathogenic effector cells but also as suppressor cells in an immune response. Here, we review the published literature regarding the known and potential inflammatory and regulatory roles of mast cells in cutaneous inflammation as well as in host defense.

## Mast Cells in Skin Inflammation During Infection

**Figure 1:**
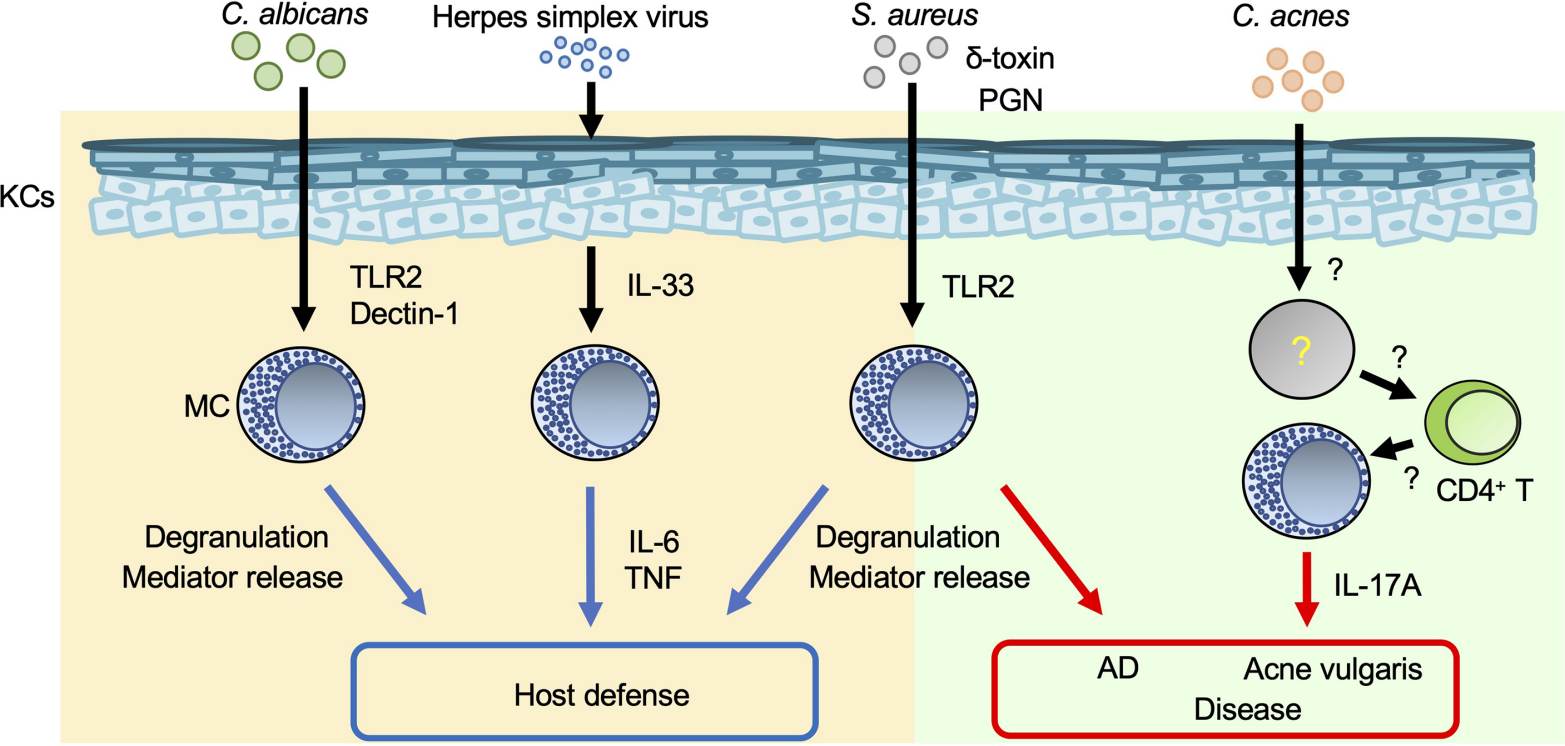
Mast cells in skin inflammation during infection. Mast cells (MCs) are important for host defense against various pathogens. On the other hand, inappropriate MC activation during infection results in aggravation of such skin diseases as AD and acne vulgaris. Blue arrows: appropriate MC activation; red arrows: excessive MC activation.

Toll-like receptors (TLRs), which are pattern-recognition receptors (PRRs), play a crucial role in host defense against various types of pathogens by recognizing pathogen-associated molecular patterns (15). Peptidoglycan (a TLR2 agonist) and lipopolysaccharide (LPS; a TLR4 agonist) are, respectively, components in the cell walls of gram-positive bacteria and mycobacteria, and of gram-negative bacteria. Poly (I:C) (a TLR3 agonist) is a mimic of viral dsRNA. These molecules have been shown to be able to induce cytokine and/or chemokine production by murine and/or human mast cells. In addition, various other peptidoglycans can induce degranulation of murine and/or human mast cells (16–18). Thus, mast cells are important for host defense against viruses and bacteria through PRRs such as TLRs. On the other hand, pathogen-derived antigens/components can promote excessive mast cell activation, resulting in exacerbation of inflammation (**Figure 1**).

*Staphylococcus (S.) aureus* is a gram-positive bacterium that causes impetigo and staphylococcal scalded skin syndrome (SSSS), primarily in young children (19, 20). Impetigo is a superficial bacterial skin infection that occurs in bullous or non-bullous form. The exfoliative toxin of *S. aureus* cleaves desmoglein 1 and causes bullous impetigo (19). Hematogenous dissemination of exotoxins from the initial site of *S. aureus* infection leads to separation of epidermal keratinocytes and detachment of the superficial epidermis in SSSS (20). *S. aureus* invades and survives in human cord blood-derived mast cells (CBMCs) after internalization (21). *S. aureus* and *S. aureus*-derived peptidoglycan induce degranulation and cytokine production by human CBMCs and mouse bone marrow-derived cultured mast cells (BMCMCs) (16, 17, 21). Mast cells degranulate in response to *S. aureus*-derived δ-toxin, contributing to increased vascular permeability in mice (22). In addition, mast cells are responsible for development of *S. aureus*-mediated skin inflammation accompanied by spongiosis, parakeratosis, and neutrophil infiltration, suggesting that *S. aureus*-stimulated mast cells exacerbate dermatitis such as atopic dermatitis (AD) (22).

Acne vulgaris is a common cutaneous disorder characterized by chronic and recurrent development of multiple inflammatory papules, pustules and nodules, mainly on the face but also on the neck, chest and back. Hyperkeratotic plugs composed of corneocytes in the lower portion of the follicular infundibulum create a new environment that impacts the microbiota and fosters proliferation of a gram-positive bacillus, *Cutibacterium acnes*. IL-17A-producing mast cells were detected in the perifollicular area of acne vulgaris lesions (23). Activated memory/effector CD4^+^ T cells induced IL-17A production by human mast cells, implying a contribution of mast cells to development of acne vulgaris (23). However, their precise role remains unclear.

A fungus, *Candida*, is part of the normal flora of the gastrointestinal tract, oral/nasal cavity, and skin of humans, but it can cause disease when host immunity is compromised or there is an imbalance in the ecological niche. Mast cells reside in various tissues that can be colonized or infected by *Candida* spp., including *Candida* (*C.) albicans* (24). A human mast cell line (HMC-1) degranulated and produced IL-8 in response to *C. albicans*, contributing to enhanced migration of neutrophils (25). Rodent mast cells can phagocytose *C. albicans* and produce nitric oxide, a reactive oxygen species, cytokines and chemokines *via* Dectin-1 and/or TLR2 (26–29). These results suggest that mast cells play a critical role in host defense against *C. albicans* infection, but their precise role in the setting needs to be elucidated.

Dermatophytes are filamentous fungi of the genera *Trichophyton*, *Microsporum*, and *Epidermophyton* that infect the skin, hair, and nails. The yeast and mycelial forms of *Malassezia* are found in the skin scales of patients with pityriasis versicolor (30). MGL_1304, derived from *Malassezia* (*M.*) *globosa*, was identified in human sweat by mass-spectrometric analysis based on the histamine-releasing activity in basophils of patients with AD (31). Serum specific IgE against MGL_1304 was higher in patients with AD and cholinergic urticaria, which is a subtype of chronic urticaria whose symptoms are evoked by sweating, than in normal controls (32). To the best of our knowledge, there are no reports of other types of urticarias that involve MGL_1304. The level of degranulation of a human mast cell line, LAD2, sensitized with sera from patients with AD was greater than with healthy control sera after stimulation with MGL_1304 (32), suggesting that MGL_1304 is a major allergen involved in the exacerbation of AD and cholinergic urticaria *via* induction of mast cell degranulation.

Herpes simplex virus (HSV) is a double-stranded DNA virus and the cause of a common viral infection of epidermal cells that is typically transmitted *via* physical contact (33, 34). HSV can be transmitted even if the source is asymptomatic, but transmission is more likely if the source is symptomatic because the viral titer is much greater when lesions are present (33). Infections by HSV types 1 and 2 are characterized by recurrent, vesicular lesions that are accompanied by pain, tingling, pruritus, and/or burning. Lesions can develop anywhere on the body but occur mainly on the lips (HSV-1) and in the genital area (HSV-2) (33). HSV infections are associated with onset of eczema herpeticum (Kaposi’s varicelliform eruption) in patients with AD (35) or erythema multiforme (36). Mast-cell-deficient *Kit^W^/Kit^W-v^* mice were susceptible to HSV-2 compared with *Kit* ^+/+^ mice (37). HSV-2 induces IL-33 production by keratinocytes, followed by activation of mast cells to produce IL-6 and tumor necrosis factor (TNF) (38), which are crucial for host defense against HSV-2 (37, 38).

## Mast Cells in Contact Hypersensitivity

**Figure 2:**
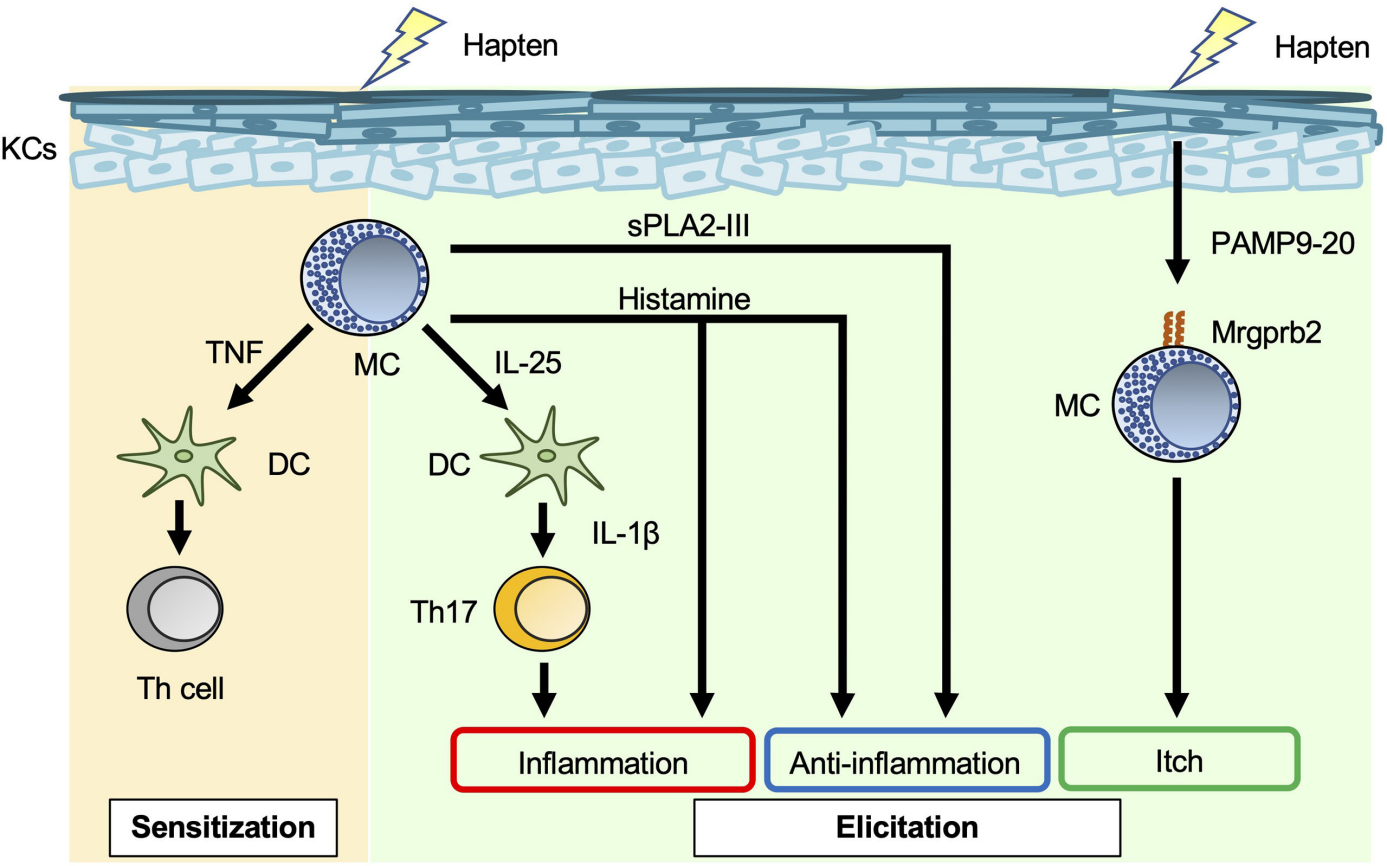
Mast cells in acute contact hypersensitivity. Mast cell (MC)-derived TNF enhances skin dendritic cell (DC) migration to draining LNs, leading to induction of hapten-specific Th-cell expansion in the sensitization phase of contact hypersensitivity (CHS). MC-derived IL-25 induces IL-1β production by dermal DCs, followed by promotion of IL-17 production by Th17 cells in the elicitation phase of CHS. Histamine has dual roles, i.e., inflammatory and anti-inflammatory, in the elicitation phase of CHS. Haptens stimulate keratinocytes (KCs) to produce PAMP9-20, a ligand for Mrgprb2. PAMP9-20 induces itch during CHS.

Allergic contact dermatitis/contact hypersensitivity (ACD/CHS) develops in response to repeated skin exposure to an allergen. Haptens are generally non-immunogenic, low-molecular-weight chemicals. When haptens are applied to the skin surface, they penetrate the stratum corneum barrier and form chemically-modified, immunogenic neo-antigens by binding with self-proteins (39). Haptens such as 2,4,6-trinitrochlorobenzene (TNCB), oxazalone, 1-fluoro-2,4-dinitrobenzene (DNFB), and fluorescein isothiocyanate (FITC) have long been used experimentally to study CHS in murine models (40). As noted in other reviews (1, 8, 41), the roles of mast cells in the development of acute and chronic CHS have differed with the experimental protocol. In some studies of acute CHS models using mast-cell-deficient and -depleted mice, mast cells (especially mast-cell-derived TNF) were responsible for development of CHS (1, 8, 41–43) (**Figure 2**). In addition, mast-cell-derived IL-25 promoted IL-1β production by dermal dendritic cells, which led to exacerbation of Th17-cell-mediated skin inflammation (44). On the other hand, mast cells were not essential for development of acute CHS in certain settings using mast-cell-deficient mice (1, 8, 41). Mast cells can also play a suppressive role in induction of acute CHS in some settings. For example, mast-cell-derived IL-5 was important for expansion of IL-10-producing regulatory B cells, which resulted in suppression of acute CHS (45). Similarly, mast-cell-derived IL-13 inhibited Th1-cell activation by suppressing IL-12 production by skin dendritic cells, which resulted in attenuation of acute CHS (46). Group III secreted phospholipase A2 (sPLA2-III; encoded by *Pla2g3*) released from immature mast cells is important for prostaglandin D2 (PGD_2_) production by fibroblasts (47). In turn, fibroblast-derived PGD_2_ causes immature mast cells to differentiate into mature mast cells (47). Mast-cell-derived sPLA2-III plays a suppressive role in development of acute CHS, but a promotive role in development of irritant-induced contact dermatitis (48).

Mast cells are a major producer of histamine, which binds to H_1_R, H_2_R, H_3_R, and H_4_R. H_1_R and H_4_R play important roles in allergic diseases, such as urticaria and asthma; H_2_R stimulates gastric acid secretion; and H_3_R plays a crucial role in the control of sleep–wake behavior (49). H_1_R antagonists are widely used to treat pruritic skin inflammation, including urticaria and AD. Mice treated with H_1_R antagonists (chlorpheniramine, oxatomide, ketotifen, mequitazine, emedastine, terfenadine and azelastine) showed attenuated acute CHS (50), whereas mice treated with H_1_R antagonists (diphenhydramine, homochlorcyclizine, cyproheptadine and cetirizine) showed normal development of acute CHS (50, 51). Mice treated with an H_2_R antagonist (cimetidine) showed augmentation of acute CHS (52). In addition, mice deficient in histidine decarboxylase, which is an enzyme involved in histamine synthesis, showed exacerbation of acute CHS (53).

Mas-related G-protein-coupled receptor X2 (MRGPRX2) mRNA is most abundant in human skin, adipose tissue, the bladder, and the colon (54). MRGPRX2 is expressed on human mast cells, and its murine ortholog, Mrgprb2, is specifically expressed on murine CTMCs (55). A new technique based on near-infrared photoimmunotherapy was recently developed for ablation of cancer cells. In that technique, photosensitizer-conjugated monoclonal antibodies specific for a cell surface marker on cancer cells are delivered to the tumor, followed by activation of cytotoxicity (thermotoxicity) by illumination (56). As an extension of that technology, photosensitizer-conjugated monoclonal antibodies specific for MRGPRX2, a cell surface marker on mast cells, were employed to reduce the number of mast cells in the skin (56). MRGPRX2/Mrgprb2 is a promiscuous receptor for cationic ligands, including substance P, compound 48/80, and pro-adrenomedullin peptide 9–20 (PAMP9–20). These ligands induce degranulation of mast cells *via* MRGPRX2/Mrgprb2 (57, 58). PAMP9–20 expression is increased in the inflamed skin lesions of patients with ACD (58). Skin thickness was similarly increased in *Mrgprb2^-/-^* mice and wild-type mice during acute CHS (58), whereas scratching behavior and the number of inflammatory cells in the skin were significantly reduced in *Mrgprb2^-/-^* mice (58). Thus, Mrgprb2-mediated mast-cell-activation is somehow involved in induction of itch during acute CHS.

**Figure 3:**
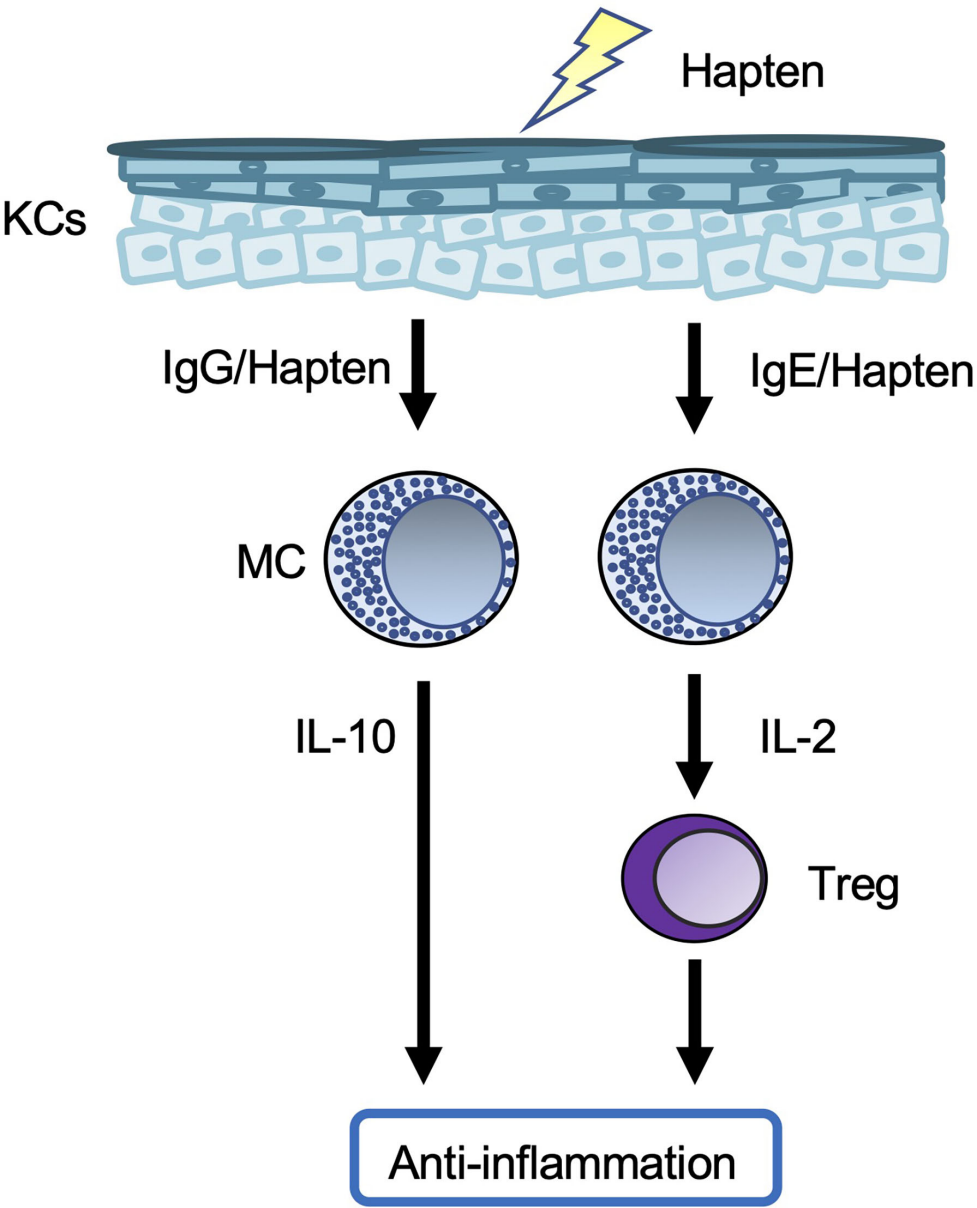
Mast cells in chronic contact hypersensitivity. In one chronic contact hypersensitivity (CHS) setting, crosslinked hapten/hapten-specific IgG complexes stimulate skin mast cells (MCs) to produce IL-10, which leads to suppression of the inflammation. In another chronic CHS setting, crosslinking of hapten/hapten-specific IgE/FcϵR1 results in production of IL-2 by splenic MCs, which leads to expansion of Tregs in the spleen. The Tregs migrate from the spleen to local skin lesions, where they suppress the inflammation.

Meanwhile, chronic CHS was induced in rodents by repeated cutaneous exposure to haptens (59–63). Mast cell-deficient/depleted mice showed increased development of chronic CHS (**Figure 3**). In one setting, hapten/hapten-specific IgG1 complexes induced IL-10 production by mast cells by binding to FcγR on the cells, resulting in IL-10-mediated suppression of chronic CHS (59, 63). In another setting, hapten/hapten-specific IgE/FcϵR1 crosslinking induced IL-2 production by mast cells (61). Mast cell-derived IL-2 enhanced regulatory T cell (Treg) expansion, followed by suppression of inflammation during chronic CHS by Tregs (61). Therefore, mast cells have dual roles as both effector cells and regulatory cells in the development of acute and/or chronic CHS induced by certain haptens.

## Mast Cells in Urticaria

Mast cell degranulation was observed in the dermis immediately below wheals in various types of inducible urticaria (64). Histamine, which is released by mast cells during IgE-mediated degranulation, is known to be crucial for the pathogenesis of urticaria (65). Increased blood levels of histamine were noted following provocation of inducible urticaria (64). In patients with chronic spontaneous urticaria, more than 200 IgEs, which recognize autoantigens including IL-24, were detected in sera (66). In addition to H_1_R antagonists, an anti-human IgE monoclonal antibody (omalizumab) was reported to provide clinical benefit for chronic spontaneous urticaria (67). Moreover, it is known that there is IgE-independent pathogenesis of chronic urticaria (64). The number and proportion of MRGPRX2-positive skin mast cells are increased in the inflamed skin lesions of patients with chronic urticaria compared with the skin of healthy control subjects (68). Intradermal administration of substance P induced greater wheal reactions in patients with chronic spontaneous urticaria than in healthy subjects (69), suggesting that substance P/MRGPRX2-mediated mast cell degranulation is an alternative pathway for induction of chronic spontaneous urticaria. In addition, increased expression of IL-25 and IL-33 on mast cells was observed in lesional skin of patients with chronic spontaneous urticaria (70). IL-25 and IL-33 can modulate many aspects of mast cell function, including proliferation and production of a variety of Th2 cytokines in chronic spontaneous urticaria (64).

## Mast Cells in Atopic Dermatitis

AD is a chronic, pruritic and inflammatory skin disease that occurs in 15–30% of children and approximately 5% of adults in industrialized nations (71). AD is characterized by barrier disruption, immunological dysfunction and elevated serum IgE. The symptoms of AD, such as recurrent dry, scaly and erythematous lesions and intense pruritus, can place an enormous burden on patients. A significant association was observed between the number of mast cells in AD skin lesions and the disease severity (assessed by the Eczema Area and Severity Index (EASI) score), although the number of mast cells was not changed by short-term treatment with topical tacrolimus (72).

Filaggrin, a filament-associated protein, is crucial for maintenance of the skin barrier (73). Mutations in the *filaggrin* gene are associated with increased prevalence of ichthyosis vulgaris and AD (73, 74). Mice with mutations in the *filaggrin* gene (*Flg^ft^* mice; also called flaky tail mice) spontaneously develop dermatitis that is accompanied by increases in the serum IgE and number of dermal mast cells, thus resembling AD (75).

Topical application of a low-calcemic vitamin-D3-analog MC903 (calcipotriol), which is widely used in the treatment of psoriasis, resulted in development of AD-like dermatitis in mice. MC903-induced dermatitis was dependent on thymic stromal lymphopoietin (TSLP), IL-25 and IL-33 in BALB/c mice (76), and on TSLP, but not IL-25 or IL-33, in C57BL/6 mice (77). Mast cells were involved in TSLP production and induction of skin inflammation in MC903-induced dermatitis (78).

Nc/Nga mice developed AD-like skin inflammation after topical application of an ointment containing *Dermatophagoides farinae* (Dfb) (79). The number of mast cells and histamine level were increased in the inflamed skin of Dfb-treated Nc/Nga mice (79). Although the frequency of scratching was decreased by application of an H4R antagonist (JNJ 7777120) to the skin of wild-type mice after intradermal histamine injection, that same antagonist was ineffective against itching and skin inflammation in Dfb-induced AD-like skin inflammation in NC/Nga mice (80).. Meanwhile, the EASI score was lower for inflammatory AD skin lesions in patients who were treated with an H_4_R antagonist (ZPL-3893787) than in those treated with a placebo (81).

Mast cells produce IL-4 and IL-13 (8). Treatment of AD patients with an anti-human IL-4Rα antibody (dupilumab) that inhibits binding of IL-4 and IL-13 to IL-4Rα improved the signs and symptoms of AD (including pruritus), anxiety and depression, as well as the quality of life, compared to placebo controls (82). However, to date, neither meta-analyses and systematic reviews of existing case series nor randomized controlled trials (RCTs) have generated concrete evidence of overall effectiveness of omalizumab for AD (83).

## Mast Cells in Psoriasis

Psoriasis is a common chronic skin disease involving systemic inflammation that leads to formation of scaly patches on the skin. The number of mast cells in pruritic lesions was greater than in non-pruritic lesions in psoriasis (84). Activated mast cells were more abundant in psoriatic lesions than in non-lesional psoriatic skin and in healthy subjects, whereas resting mast cells were almost entirely absent in psoriatic skin lesions (85). Importantly, the proportion of resting mast cells gradually normalized in lesional psoriatic skin during etanercept (a TNF inhibitor) therapy (85). These results suggest that mast cells may be involved in the pathogenesis of psoriasis.

**Figure 4:**
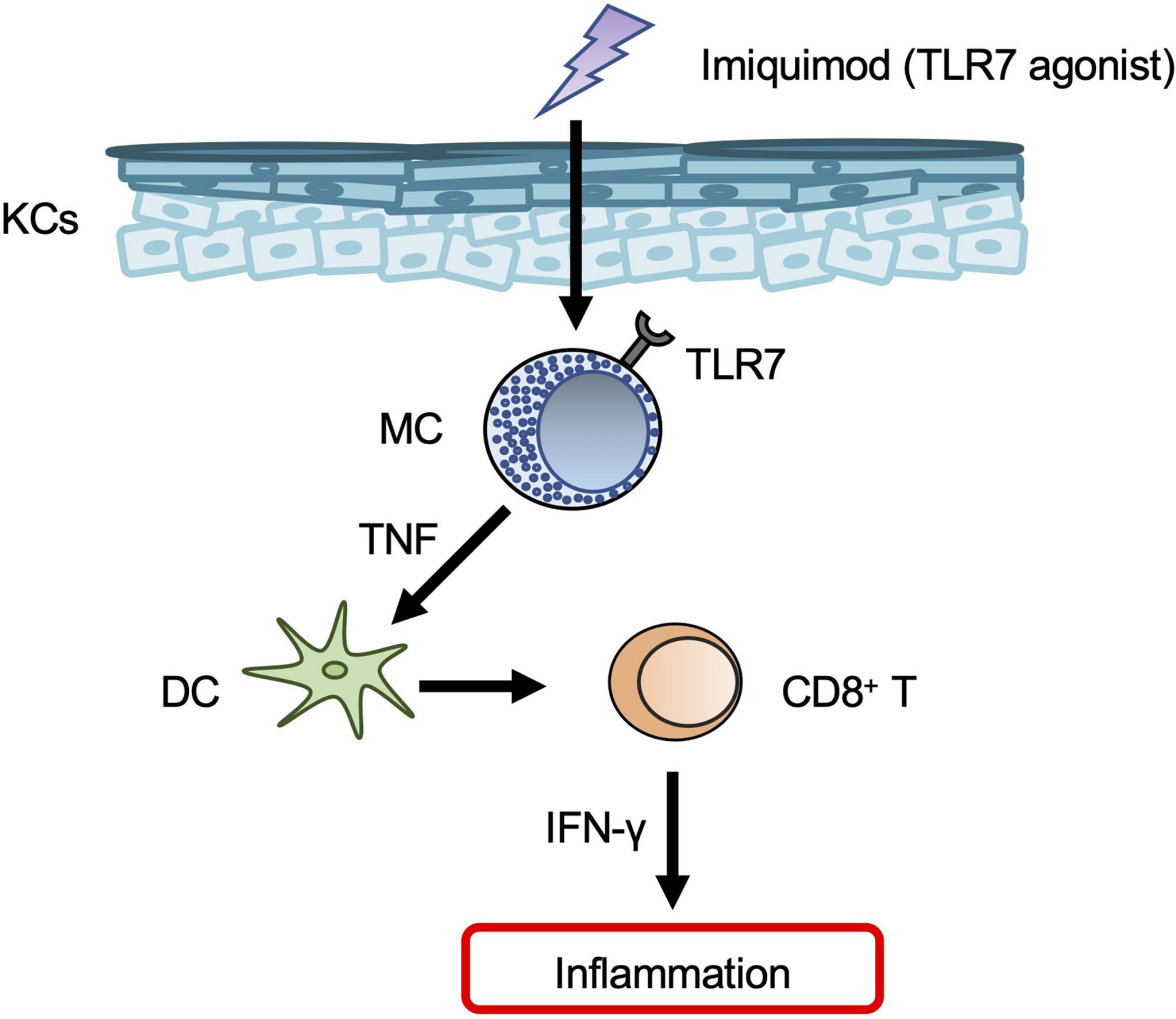
Mast cells in psoriasis. Mast cells (MCs) produce TNF in response to imiquimod *via* TLR7, and TNF then promotes dendritic cell (DC) migration from the skin to draining LNs. The migrated DCs induce CD8^+^ T-cell expansion in the peripheral blood.

Topical application of imiquimod — a TLR7 agonist that is widely used to treat genital warts and actinic keratosis — resulted in development of psoriasis-like dermatitis in mice (86). However, it should be noted that the dermatitis induced in humans by topical imiquimod resembled contact dermatitis rather than psoriasis (87). Expression of *Tlr7* mRNA was constitutively observed and increased in mouse BMCMCs in response to imiquimod, and TLR7 on mast cells was responsible for development of imiquimod-induced dermatitis in mice (88). In those same mice, mast cell activation by imiquimod *via* TLR7 led to TNF production, which in turn promoted skin dendritic cell migration (88) (**Figure 4**). Imiquimod can induce degranulation of mast cells in humans and mice dependent on human MRGPRX2 and mouse Mrgprb2 (89), although it remains unclear whether imiquimod can bind to MRGPRX2/Mrgprb2. Mrgprb2-dependent mast cell degranulation is crucial for development of imiquimod-induced dermatitis in mice (89).

## Mast Cells in Collagen Synthesis (Wound Healing and Fibrosis)

**Figure 5:**
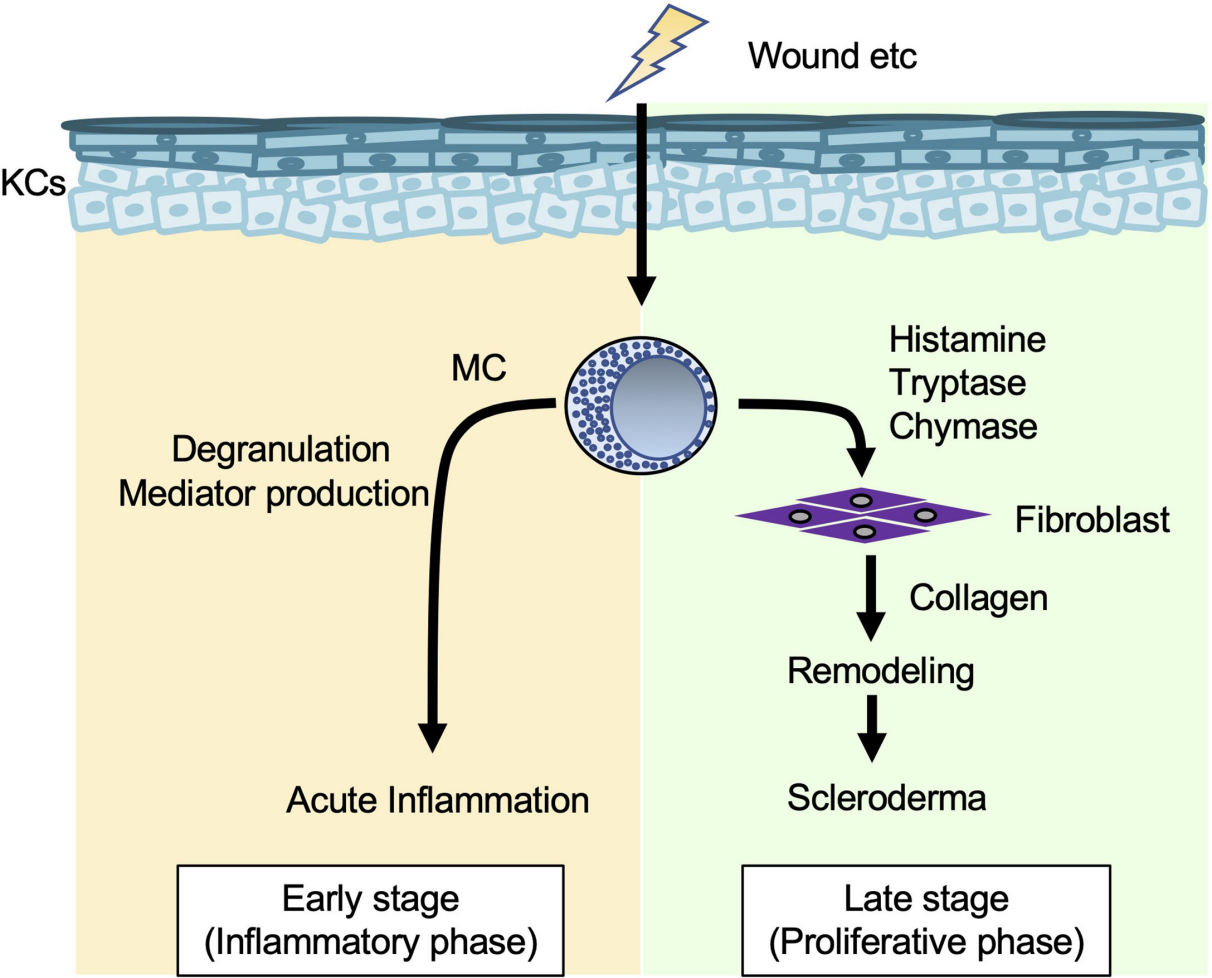
Mast cells in collagen synthesis (wound healing and fibrosis). When the skin is wounded, mast cells (MCs) degranulate and release various mediators that enhance vascular permeability and recruitment of inflammatory cells to injured sites in the early stage (inflammatory phase). In addition, MC-derived histamine, tryptase and chymase induce fibroblasts to produce collagen, which is involved in tissue remodeling and development of scleroderma in the late stage (proliferative phase).

Mast cells activated by tissue injury regulated various phases of skin repair (90). In mice 0.5~1 hour after wounding, the number of degranulated mast cells and the level of vascular permeability were most prominently increased in areas directly adjacent to the wounded skin (91). Moreover, mast cells were involved in neutrophil influx and wound healing in the wounded skin of mice at 12 hours and two to six days after wounding, respectively (91). Thus, mast cells appear to be important for induction of inflammation by increasing vascular permeability and recruiting inflammatory cells to wound sites. Scar width was significantly smaller in mast-cell-deficient *Kit^W/W-v^* mice than in *Kit^+/+^* mice at seven and 10 days after wounding (92). In addition, activated mast cells promoted fibroblast expansion (93). These data suggest that mast cells are involved in collagen deposition by activating fibroblasts during remodeling (**Figure 5**).

Systemic sclerosis (scleroderma) is a systemic autoimmune connective tissue disorder characterized by vascular dysfunction and progressive fibrosis of the skin and internal organs, such as the lung and kidney. Mast cell density (mast cells/mm^2^) in the papillary and reticular dermis was significantly greater in patients with early progressive systemic sclerosis than in control subjects (94). Histamine and tryptase each enhanced proliferation and collagen synthesis in human skin fibroblasts (95). In aged tight-skin mice, which develop an inherited fibrotic disease resembling scleroderma, mast cells and chymase were responsible for augmentation of fibrosis (96, 97).

Mast cells were detected in lichen planopilaris (LPP), a type of scarring hair loss (cicatricial alopecia) characterized by lymphocytic infiltration in the upper portion of hair follicles (98). The number of IL-17A-positive mast cells was increased in LPP lesions compared with the normal scalp (98). IL-17R is expressed exclusively in follicular epithelial cells in LPP lesions. These observations suggest that mast-cell-derived IL-17A might somehow be involved in the pathogenesis of LPP *via* IL-17R on follicular epithelial cells.

## Mast Cells in Skin Allografts and Graft-Versus-Host Disease (GVHD)

Long-term acceptance of skin allografts was enabled in mice by injection of anti-CD154 blocking antibody together with allogeneic cells (a process known as donor-specific transfusion; DST) (99). Tregs were important for tolerance to alloantigens in mice (100), and mast cells were increased in the skin allografts of DST-treated mice (99). In addition, in that DST model, Treg-derived IL-9 induced mast cell accumulation and activation in skin allografts, and the accumulated cells suppressed CD8+ T cell-mediated allograft rejection (99).

**Figure 6:**
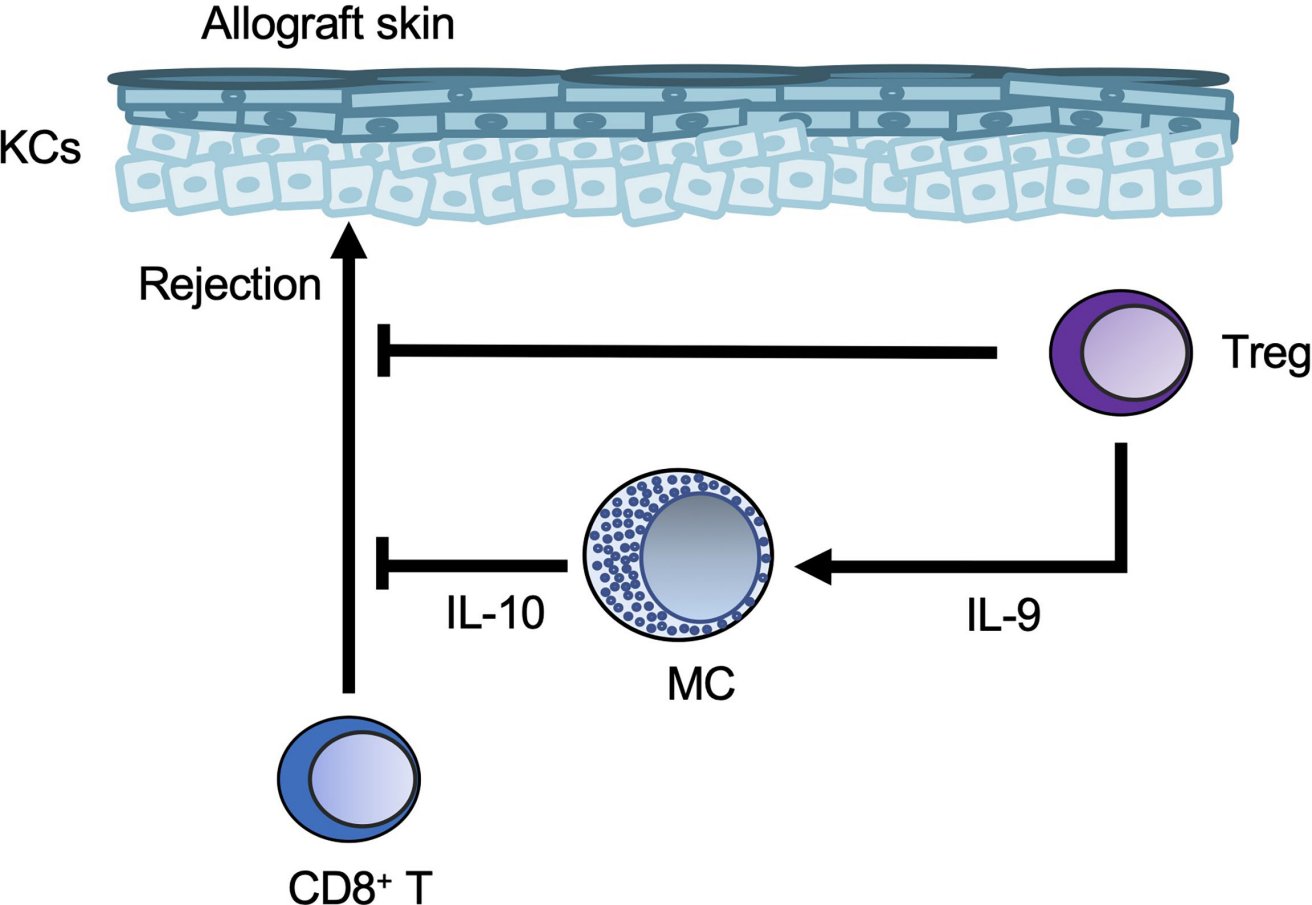
Mast cells in skin allografts. Regulatory T-cell (Treg-)derived IL-9 induces mast cell (MC) accumulation and activation in skin allografts. The Tregs and MCs then suppress CD8+ T-cell-mediated allograft rejection.

A major side effect of allogeneic hematopoietic stem cell transplantation is graft-versus-host disease (GVHD), in which donor lymphocytes attack the recipient’s body as non-self tissues. The number of mast cells was increased in the skin of patients with more severe acute GVHD (101). In GVHD induced by transplantation of CD8^+^ T cells and T cell-depleted bone marrow cells from C3H.SW mice, the survival rate was significantly higher for irradiated WBB6F1-*Kit^W/W-v^* mice than for irradiated WBB6F1-*Kit^+/+^* mice (102). The density of dyskeratotic cells was significantly lower in WBB6F1-*Kit^W/W-v^* mice than in WBB6F1-*Kit^+/+^* mice at 14, 21, and 28 days after transplantation, suggesting that mast cells act as effector cells in the development of acute GVHD (102) (**Figure 6**).

On the other hand, development of acute GVHD induced by intravenous injection of either T cell-depleted bone marrow from C57BL/6 mice or CD4^+^ and CD8^+^ T cells from FVB mice was significantly greater in irradiated C57BL/6-*Kit^W-sh/W-sh^* mice than in irradiated C57BL/6 wild-type mice (103). These GVHD reactions were resolved in C57BL/6-*Kit^W-sh/W-sh^* mice engrafted with wild-type BMCMCs, but not *Il10^-/-^* BMCMCs (103). These results indicate that mast-cell-derived IL-10 plays an important role in the inhibition of acute GVHD caused by MHC antigen mismatch.

## Conclusion

**Figure 7:**
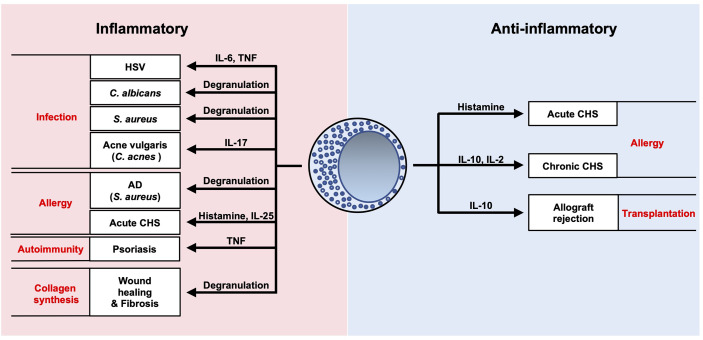
Summary of roles of mast cells in cutaneous diseases. Mast cells are potential sources of inflammatory and/or regulatory mediators during development of various cutaneous infections and diseases.

Mast cells act as potential sources of inflammatory and/or regulatory mediators during development of various cutaneous infections and diseases (**Figure 7**). Considerable progress has been made in our understanding of these immune cells in recent years. Further elucidation of the complex interactions of mast cells will potentially lead to novel clinical approaches for various pathological conditions.

## Author Contributions

TN designed and wrote the manuscript. KH and SN reviewed and revised the manuscript prior to submission. All authors contributed to the article and approved the submitted version.

## Funding

This work was supported by a Grant-in-Aid for Scientific Research (B)(21H02963 to SN) from the Japan Society for the Promotion of Science, and Precursory Research for Embryonic Science and Technology, Japan Science and Technology Agency (JPMJPR18H6 to SN).

## Conflict of Interest

The authors declare that the research was conducted in the absence of any commercial or financial relationships that could be construed as a potential conflict of interest.

## Publisher’s Note

All claims expressed in this article are solely those of the authors and do not necessarily represent those of their affiliated organizations, or those of the publisher, the editors and the reviewers. Any product that may be evaluated in this article, or claim that may be made by its manufacturer, is not guaranteed or endorsed by the publisher.

## Abbreviations

ACD, allergic contact dermatitis; AD, atopic dermatitis; BMCMCs, bone marrow-derived cultured mast cells; CBMCs, cord blood-derived mast cells; CHS, contact hypersensitivity; CTMC, connective tissue-type mast cell; Dfb, *Dermatophagoides farina*; DNFB, 1-fluoro-2,4-dinitrobenzene; DST, donor-specific transfusion; EASI, Eczema Area and Severity Index; FITC, fluorescein isothiocyanate; GVHD, graft-versus-host disease; HSV, herpes simplex virus; IL, interleukin; Ig, immunoglobulin; LPP, lichen planopilaris; LPS, lipopolysaccharide; MCPs, mast cell progenitors; MMC, mucosal mast cell; MRGPRX2, mas-related G-protein-coupled receptor X2; NGF, nerve growth factor; PAMP9–20, pro-adrenomedullin peptide 9–20; PGD_2_, prostaglandin D2; PRRs, pattern-recognition receptors; RCTs, randomized controlled trials; SCF, stem cell factor; sPLA2-III, group III secreted phospholipase A2; SSSS, staphylococcal scalded skin syndrome; TGF, transforming growth factor; TLR, toll-like receptors; TNCB, 2,4,6-trinitrochlorobenzene; TNF, tumor necrosis factor; Treg, regulatory T cell; TSLP, thymic stromal lymphopoietin.
